# Trends and serotype distribution of human Salmonella strains in central Italy (2015-2021)

**DOI:** 10.1093/eurpub/ckac130.193

**Published:** 2022-10-25

**Authors:** V Russini, C Lucarelli, MG Marrocco, S Owczarek, L Villa, ML De Marchis, S Bilei

**Affiliations:** 1 Food Microbiology Unit, Istituto Zooprofilattico Sperimentale del Lazio e Toscana, Rome, Italy; 2 Department of Infectious Diseases, Istituto Superiore di Sanità, Rome, Italy

## Abstract

**Introduction:**

Salmonella enterica (S.) is one of the most common agents of foodborne infections and a risk for children, elder people and immunocompromised patients. S. is the first cause of foodborne outbreaks in the EU, the majority being caused by S. Enteritidis. We report S. serovars prevalence and trends in clinical isolates in central Italy from 2015 to 2021.

**Methods:**

S. strains of patients from Lazio and Tuscany regions isolated by hospitals and private laboratories were sent to the Regional Reference Centre for Pathogenic Enterobacteria (CREP) at IZSLT (Rome) for serotyping. All metadata and a selection of isolates were shared with ISS according to the National Surveillance Enter-Net Italia program.

**Results:**

A total of 2395 strains were collected from 2015 to 2021, with a mean value of 342 strains per year. Notably, reported cases did not decrease during the pandemic in 2020. A total of 116 different serovars were identified. The most common ones were S. Typhimurium var. monophasic, which increased from 2015 to 2021, S. Enteritidis, which peaked in 2018 doubling its average, and S. Typhimurium with a reverse trend compared to the monophasic variant, followed by S. Infantis, S. Napoli and S. Derby. Afterwards, S. Brandenburg showed a constant increase (from 2 cases in 2015 to 18 cases in 2021). S. Strathcona showed a significant peak during 2019 with 23 cases, correlated to a European reported outbreak. The average age of patients was stable (mean 28.5, median 12.8), except for an increase in 2021 (mean 35.2, median 27).

**Conclusions:**

The results show a substantial agreement between data collected in central Italy and national trends. The occurrence of cases per year is stable. The serovars prevalence does not agree with the prevalence found in EU, in particular concerning S. Typhimurium var. monophasic frequency. Further investigations are needed to explain the significant increment of patients’ mean age in 2021.

**Key messages:**

- This kind of studies provide a detailed picture of the S. serotypes actually relevant to human health and could be used to improve and update food safety regulations and monitoring programs.
- Surveillance and digital reporting, even retrospective, are pivotal tool for long-term monitoring, outbreaks investigation and research.

